# Livelihood activities, human mobility, and risk of malaria infection in elimination settings: a case–control study

**DOI:** 10.1186/s12936-023-04470-0

**Published:** 2023-02-13

**Authors:** Shaymaa A. Abdalal, Joshua Yukich, Katherine Andrinopoulos, Maimonah Alghanmi, Majed H. Wakid, Ayat Zawawi, Steve Harakeh, Sarah A. Altwaim, Hattan Gattan, Fadi Baakdah, Mahmoud A. Gaddoury, Hatoon A. Niyazi, Jawahir A. Mokhtar, Mohammed H. Alruhaili, Isra Alsaady, Rowa Alhabbab, Mohamed Alfaleh, Anwar M. Hashem, Ziab Zakey Alahmadey, Joseph Keating

**Affiliations:** 1 Department of Medical Microbiology and Parasitology, Faculty of Medicine, King Abdulaziz University, Jeddah, Saudi Arabia; 2 School of Public Health and Tropical Medicine, Tulane University, New Orleans, LA USA; 3 Vaccines and Immunotherapy Unit, King Fahd Medical Research Centre, King Abdulaziz University, Jeddah, Saudi Arabia; 4 Department of Medical Laboratory Sciences, Faculty of Applied Medical Sciences, King Abdulaziz University, Jeddah, Saudi Arabia; 5 Special Infectious Agents Unit, King Fahd Medical Research Centre, King Abdulaziz University, Jeddah, Saudi Arabia; 6 King Fahd Medical Research Centre, King Abdulaziz University, Jeddah, Saudi Arabia; 7 Department of Community Medicine, Faculty of Medicine, King Abdulaziz University, Jeddah, Saudi Arabia; 8 Department of Pharmaceutics, Faculty of Pharmacy, King Abdulaziz University, Jeddah, 21589 Saudi Arabia; 9 Microbiology and Serology Departments, Al-Ansar Hospital, Ministry of Health, Medina, Saudi Arabia

**Keywords:** Malaria, Border malaria, Saudi Arabia, Human movements

## Abstract

**Background:**

Livelihood activities and human movements participate in the epidemiology of vector-borne diseases and influence malaria risk in elimination settings. In Saudi Arabia, where malaria transmission intensity varies geographically, it is vital to understand the components driving transmission within specific areas. In addition, shared social, behavioural, and occupational characteristics within communities may provoke the risk of malaria infection. This study aims to understand the relationship between human mobility, livelihood activities, and the risk of malaria infection in the border region of Jazan to facilitate further strategic malaria interventions. In addition, the study will complement and reinforce the existing efforts to eliminate malaria on the Saudi and Yemen border by providing a deeper understanding of human movement and livelihood activities.

**Methods:**

An unmatched case–control study was conducted. A total of 261 participants were recruited for the study, including 81 cases of confirmed malaria through rapid diagnostic tests (RDTs) and microscopy and 180 controls in the Baish Governorate in Jazan Provinces, Saudi Arabia. Individuals who received malaria tests were interviewed regarding their livelihood activities and recent movement (travel history). A questionnaire was administered, and the data was captured electronically. STATA software version 16 was used to analyse the data. Bivariate and multivariate analyses were conducted to determine if engaging in agricultural activities such as farming and animal husbandry, recent travel history outside of the home village within the last 30 days and participating in spiritual gatherings were related to malaria infection status.

**Results:**

A logistical regression model was used to investigate components associated with malaria infection. After adjusting several confounding factors, individuals who reported travelling away from their home village in the last 30 days OR 11.5 (95% CI 4.43–29.9), and those who attended a seasonal night spiritual gathering OR 3.04 (95% CI 1.10–8.42), involved in animal husbandry OR 2.52 (95% CI 1.10–5.82), and identified as male OR 4.57 (95% CI 1.43–14.7), were more likely to test positive for malaria infection.

**Conclusion:**

Human movement and livelihood activities, especially at nighttime, should be considered malaria risk factors in malaria elimination settings, mainly when the targeted area is limited to a confined borderland area.

## Background

Malaria continues to be an overwhelming global health problem, with an estimated 247 million infections each year and around 619 000 deaths in 2021. Saudi Arabia has successfully reduced the malaria incidence rate below 5/1000 per year, and the country successfully entered the elimination phase in 2007 [1, 2]. The country is considered malaria-free with the exception of Aseer and Jazan region in the South, where transmission is confined to small foci within the border area. Over the past 15 years, a large rapid scale-up of malaria control activities targeting high-risk areas for sustained preventative measures like long-lasting insecticidal nets (LLINs) and indoor residual spraying (IRS), timely management of infection through rapid diagnostic tests (RDTs), and using artemisinin-based combination therapy (ACT); individual case follow–up and reactive case detection and, active case detection at borders with screening and treatment [3].

Despite Saudi Arabia’s goal of malaria elimination by 2020, in the last five years, the country’s progress has reduced, and current elimination efforts failed to contain malaria at the southern border shared with highly endemic areas of war–torn Yemen [4]. Malaria transmission in Jazan region presents a real challenge for malaria surveillance [5]. This is because malaria infection is disproportionally clustered in subpopulations that live within the border region, work in agriculture, and share social, behavioural, and occupational characteristics [4, 6, 7]. Individuals who are active at dusk due to their occupation (farmers, truck drivers, and watchmen), their cultural practices (e.g., group prayer, weddings, watching football games), or periodic movement for other reasons such as economic reasons, business trips, seasonal workers, smuggling, and refugees are not benefiting from using ITNs or IRS [8–11].

Correspondingly, in other elimination settings, agricultural livelihood activities, travelling, and outdoor social gatherings are linked to increased risk of malaria infection due to prolonged contact with mosquito vectors where standard preventive interventions may not be particularly effective [12–15].

Several studies have shown that human movement contributes to malaria transmission [16–18]. Population movement between areas of high- and low-risk, both at macro–scale across an international border or regional level [19–22] and micro–scales community, short distance, different times of the day [23–29], exposes the population to different environmental conditions and mosquito biting intensity [30]. Additionally, human movement associated with commerce and trade through shipping industries and ports has led to the global spread of other vector–borne diseases [31, 32].

Notably, movements related to agriculture-like large-scale migration of labourers searching for economic opportunities play role in malaria epidemiology [33, 34]. For example, in the early 1950s, malaria was nearly eliminated in Swaziland; however, agricultural development with numerous irrigation systems created excellent conditions for malaria [35, 36]. To date, agricultural labourers remain a high-risk group for malaria infection from importation cases even though Swaziland is progressing towards malaria elimination, and there is very little locally acquired malaria [35]. Furthermore, vector population expanded due to the increase in agricultural use of irrigation systems, and mosquitoes contact with infected people from neighbouring endemic areas of Mozambique, which led to a resurgence of malaria in Swaziland [36].

Equally important are the Islamic spiritual practices and their timing and place carried out routinely in any Muslim community and often overlooked as possible transmission settings. Three routine spiritual gathering activities occur, when malaria vectors *Anopheles arabiensis* are active [37]. Moreover, extended changes to sleeping and activity patterns occur throughout Ramadan; the Islamic holy month were Muslims fast from sunrise until sunset. Increases in nighttime activities include group prayers and spending some or all the night outdoors. Therefore, a clear understanding of human behaviour is needed to identify risk factors and proper prevention measures.

Due to the limitation in published studies focused on malaria in Jazan, the aim of this study is to observe key risk factors such as travel history, agricultural activities, and spiritual gatherings as potential malaria transmission and infection settings in agricultural areas. Further study is needed to guide strategic planning for future malaria elimination activities to reach a malaria-free country and zero local cases.

## Methods

### Study design

A community-based, unmatched case–control study was conducted in the Baish governorate in Jazan region of Saudi Arabia. Data were collected from case investigations/reactive case detection (RCD) carried out by malaria and vector control programme in Baish Malaria Centres in Jazan Provinces, Saudi Arabia. The study utilized the existing resources of the programme and recruited trained staff from the malaria elimination programme.

*Cases* were defined as subjects who reported to the malaria centre in Baish with a confirmed positive thick and/or thin blood smear or rapid diagnostic test high risk (RDT) from the local hospital, primary health centre, or malaria centre. All case investigations/reactive case detection (RCD) carried out by the study-trained staff in Baish malaria elimination centre during the study period were included. Controls were randomly selected from the community members living near clinical cases (index cases) within a radius of 500 m village. However, they did not live in the same household, and a random selection will take place; every third person who will be screened for malaria during the RCD who test malaria negative will be eligible to join the study and asked to join the study.

*Controls* are defined as subjects screened during the case investigation whose RDTs were negative for *Plasmodium* infection (additionally, thin blood smear were reviewed later for confirmation) and lived in the same village but do not live in the same household or work on the same farm. The selected eligible candidates were consented to join the study and asked to answer the questionnaire.

### Study site

**Fig. 1:**
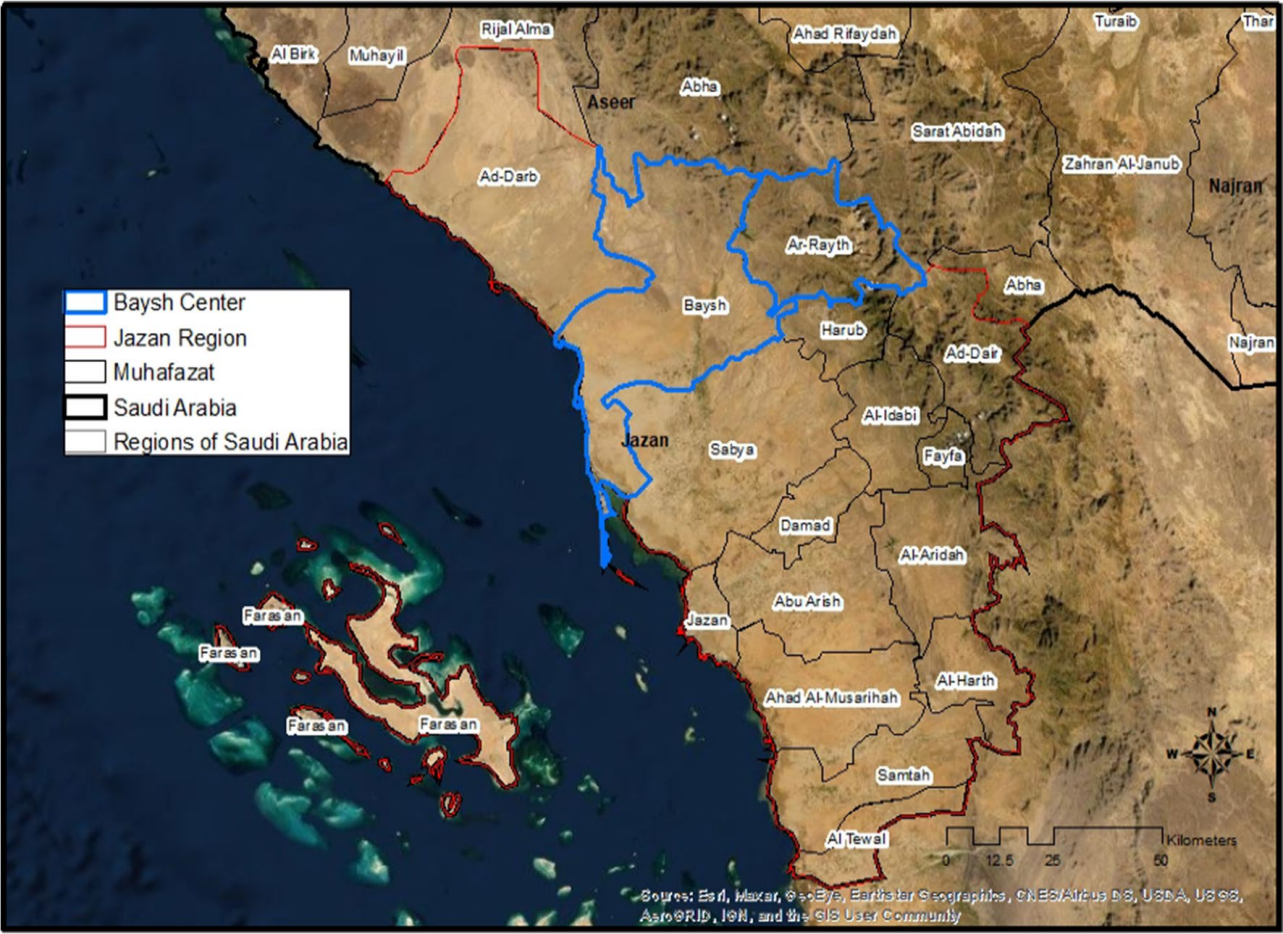
Baish Malaria centre catchment area, by Carter B, licensed under CC by 2.0

Baish malaria control centre is one of the nine malaria control centers in Jazan region. It serves as a head office for two governorates Baish and Al-Rayth. It oversees five peripheral malaria and vector control facilities that all together deliver malaria control activities in one of the region’s most significant agricultural areas, occupying 23% of Jazan total area (an area of approximately 7500 hectares (ha) of agriculture area) (Fig. 1) [38]. Baish is in the valley of Baish in Tihamah lowland plain at 400–600 m elevation above sea level near the Red Sea coast [38]. The valley contains more than 90 water streams, where more than 455 villages are scattered, prone to flooding during the rainy season [14]. It is also where the tallest dam in Saudi Arabia was constructed at 106 m (348 ft.) in height. It is used for irrigation and groundwater recharge to supply the surrounding agriculture [14]. The current population is 77,442 people [39]. Residents are mainly engaged in agricultural activities such as growing coffee, millet, corn, maize, mangoes, bananas, fruits, and vegetables; raising domestic livestock (sheep, goats, camels, cows, and poultry); and handicraft work such as ceramics, pottery, and leather goods [40]. Malaria and vector control centre is in Alhaqo area, the famous historic land route that connects Yemen–Jazan–Jeddah. Travel to Jazan city is possible through well-paved roads. However, the network between areas and surrounding villages is a mix of paved and unpaved roads.

Malaria transmission is seasonal during the rainy season (October–April). Malaria transmission peaked in January [41], mainly during the winter. *Plasmodium falciparum* predominates malaria transmission [5]. The primary malaria vector is *An. arabiensis*, the only positive vector for malaria in southern Saudi Arabia [42] with a sporozoite rate (0–0.7%) [5]. Population vector presents in low density during autumn through spring and peaks during winter following the rainy season [5]. The natural habitat for *An. arabiensis* are valleys and rain pools, but it also can be found in domestic water containers and rock pools (shallow water with pH7–9) [43]. Adult mosquitoes exhibit both endophagic and exophagic biting behaviour [44]. Interestingly in a study by Al-sheikh et al., only 40% of bloodmeals were of human origin [45, 46]. *Anopheles arabiensis* feeds and rests both indoors and outdoors. For this reason, current vector control methods targeting households, such as indoor residual spraying and insecticide-treated nets, or long-lasting insecticidal nets, may not sufficiently mitigate transmission events that occur in outdoor settings [47–50].

### Inclusion and exclusion criteria

*Inclusion* criteria for the study were individuals who are reported to Baish malaria centres in Jazan Provinces during two rainy seasons from (Aug 2017–Jan 2018) to (Aug 2018–Jan 2019) dates RDT-confirmed malaria cases were successfully traced to their households and enrolled in the study. Saudi Arabia and are part of case investigations/reactive case detection (RCD), age ten years and older, both male and female with history of confirmed malaria infection and received a Giemsa-stained thick and thin blood smear or RDT for malaria diagnosis.

*Exclusion* criteria for the study were: individuals under 10 years old, have history of malaria in the last 30 days (excluding the current episode), used malaria chemoprophylaxis or treatment in the last 30 days, or were residents of Jazan for less than 2 weeks [51].

### Sample size determination and recruitment

Sample size calculations were conducted in Open Epi, Version 3. The study was powered to detect an odds ratio of 2.0 with two-sided confidence level 95% and 80% power (chance of detection) on the measure of a night spent away from the home town in the last month, assuming that 20% of those who were not infected with any parasite species had travelled overnight in the past month, and a ratio of control to case 1:4. In total, a sample size of 484 individuals was needed; these consisted of 97 cases (positive persons for malaria infection) and 387 controls (negative persons for malaria infection).

### Data collection

Participants were recruited over a 20-week period during case investigations/reactive case detection (RCD) that was carried out by Baish malaria centre by personnel fluent in the Arabic and English language. After obtaining informed consent, participants were interviewed using an Android tablet to fill a pre-tested questionnaire using Open Data Kit (ODK).

ODK an extensible, open-source suite of tools designed to build information services. ODK currently provides four tools to this end: collect, Aggregate, Voice, and Build. Collect is a mobile platform that renders application logic and supports the manipulation of data. Aggregate provides a "click-to-deploy" server that supports data storage and transfer in the "cloud" or on local servers. Designed to be used together or independently, ODK core tools build on existing open standards and are supported by an open-source community that has contributed additional tools.

Data were gathered on socio–demographics and household risk factors including travel history (where did they travel international or domestic, length of the travel, reason for travel, known malaria active transmission foci’s), bed net use, agricultural activities, and spiritual gathering (see full questionnaire in appendix C). All confirmed malaria cases were used as a case. Controls who were randomly enrolled from the same community but not from the same household screened for malaria infection using RDTs, for the purpose of this study, and tested negative for malaria infection. Completed forms were uploaded to ODK aggregate database when internet was available. Data were converted to STATA 16.0 (Stata Corporation, Collage Station, TX, USA) format for analysis.

### Data analysis

Data analysis was done using STATA version 16 (Stata Corporation, College Station, TX, USA). Descriptive statistics was used to summarize independent variables and demographics. Bivariate analysis was conducted to assess the relationship between individual characteristics and the outcome of malaria infection. Chi‐square (*χ*^2^), and Fisher’s test was used (for small-sized samples) to assess the differences in bivariate outcome. A simple wealth index was created using binary variables for durable asset possession using principal component analysis (PCA) [52, 53]. The study population was then categorized into wealth quartiles [54]. Ordinal logistic regression was used to identify factors associated with the malaria infection. Factors tested include travel outside hometown in the last 30 days, currently working on animal husbandry, currently working on farming, regular attendance of spiritual gathering, and attendance of Ramadan spiritual gathering. The model also controlled for the following socio-demographic variables: age of the respondent, gender, wealth and use of bed net.

### Ethical approval

Institutional Review Board (IRB) approval was obtained from Tulane University in New Orleans, Louisiana, USA and the IRB of Training and Scholarship Administration (TSA) in the Ministry of Health Jazan, Saudi Arabia.

## Results

### Description of study sample

A total of 261 participants were successfully recruited for the study: 81 RDT- confirmed malaria cases were successfully traced to their household or working place enrolled in the study. It was not known the precise number of reported cases that were successfully traced enrolled in the study due to many filed challenges, such as political unrest, flooding, and other logistical reasons. Similarly, 180 controls were enrolled randomly from the community. Of the 81 infected participants, 71 (88%) and 10 (12%) were diagnosed with *P. falciparum* or *Plasmodium vivax*, respectively.

The participants were more likely to be male (56%). The age of study participants ranged from 10 to 80 years (median age 28 IQR 19–37 years). The demographic profile, livelihood activities and travel history of cases and control are summarized (Table 1). Although Baish Region is mainly a rural area, only 19% reported to be working full-time in agriculture; locals might not be engaged in agricultural activity full time or as primary occupation they are highly involved in the family-owned farms or animal husbandry. Only 23% of the participants owned animals, though the area is well known for breeding domestic animals especially sheep, and goats.

**Table 1:** Basic description of the study sample among persons in Baish District, August 2017–January 2019

	Positive (case)		Negative (control)		Total	
	N = 81	(%)	N = 180	(%)	N = 261	(%)
Sex*						
Male	59	(73.00)	86	(5.00)	145	(44.00)
Female	22	(27.00)	94	(52.00)	116	(56.00)
Occupation						
Agriculture	18	(22.00)	30	(17.00)	48	(18.00)
Non-Agricultural	20	(25.00)	32	(18.00)	52	(20.00)
Unemployed	43	(53.00)	118	(65.00)	161	(62.00)
Age [year; median (IQR)]	27	–	28	–	28	–
IQR 25–75%	(19–34)		(19–38)		(19–34)	
Washing machine*						
Yes	48	(59.26)	141	(78.33)	189	(72.41)
No	33	(40.74)	39	(21.67)	72	(27.59)
Mobile						
Yes	64	(79.00)	150	(83.33)	214	(82.00)
No	17	(21.00)	30	(16.67)	47	(18.00)
Travelled overnight*						
Yes	27	(33.33)	7	(3.89)	34	(13.03)
No	54	(66.67)	173	(96.11)	227	(86.97)
Work in farm*						
Yes	22	(27.16)	27	(15.00)	49	(18.77)
No	59	(72.84)	153	(85.00)	212	(81.23)
Work in animal husbandry*						
Yes	24	(29.63)	23	(12.78)	47	(18.00)
No	57	(70.37)	157	(87.22)	214	(81.00)
Daily prayer*						
Yes	51	(63)	87	(48.33)	138	(52.87)
No	30	(37.00)	93	(51.67)	123	(47.13)
Ramadan prayer*						
Yes	62	(76.54)	111	(61.67)	173	(66.28)
No	19	(23.46)	69	(38.33)	88	(33.72)
Bed net ownership						
Yes	6	(7.41)	15	(8.33)	21	(08.05)
No	75	(92.59)	165	(91.67)	240	(91.95)
Slept under the bed net the night before						
Yes	1	(1.23)	11	(06.11)	12	(4.60)
No	80	(98.77)	169	(93.89)	249	(95.40)

Significant difference between case and controls at the 5% level, using a ${\upchi }^{2}$ (Fischer’s Exact) test

*P-value < 0.05

Household structure was self-reported, seventy nine percent (79%) of participants reported that their house roof was made of reinforced concrete and metal, while 87% indicated that flooring was mainly tiles, 75% had windows. However, only 49% reported having screened windows.

The study sample size was not reached due to logistical reasons. Nevertheless, the two-tail power test with alpha = 0.05, history of travel in control = 33% and history of travel in cases = 66% and total sample size of 281 was included. The new power calculated = 0.59 or 59%. (Post hoc analysis).

### Malaria risk factors

#### Agricultural activities (animal husbandry and farming)

19% (49/261) of the study participants were primarily farmers who worked on a farm, and all were male. The most frequently grown crops were vegetables followed by fruits. Additionally, 26% (12/49) of the farmers reported travelling in the last 30 days. Farming was significantly associated with being diagnosed with malaria in the study population before adjustments UOR = 2.11 (95% CI: 1.11–3.99).

Eighteen percent (47/261) of the study participants were involved in animal husbandry, majority were male 85% (40/47) and only seven female participants reported involvement in animal rearing. The animals were mainly sheep, followed by goat and chicken. Animal husbandry was significantly associated with being diagnosed with malaria in this population before adjustment UOR = 2.87 (95% CI 1.51–5.49).

#### Travel history

More than two-thirds of the reported malaria cases had a travel history that can be traced to documented active transmission foci in the last five years. Thirty–four participants (13%) reported travelling outside their home village and staying for at least one night in the previous 30 days. This group of participants reported a total of 257 days of overnight trips. Among those reporting an overnight trip, the median length of stay was five days (interquartile range 1–29). Travel was more common among male participants than among female (76% vs. 24%). Most trips were made locally within the Baish governorate, followed by other governorates within Jazan region (27/34). Seven trips were to international locations: Four trips to Ethiopia, followed by two to Yemen and one to India. The most common reason for travel domestically and internationally was to visit family or friends (41%) followed by business (24%). Yet, 29.41% preferred not to disclose their reason for travel. Persons reporting travel in the last 30 days have a 12.40 greater odds of malaria infection than those who did not travel.

#### Bed net ownership and use

Twenty-one participant from the total study population reported owning a bed net (8%). Of those reporting owning a bed net, only (12/21) 57% slept under the bed net the night before. Also, of those who reported owning a bed net, 40% (8/20) reported that they do not know if the bed net was treated, while 60% (12/20) reported owning a treated bed net. Owners of a bed net were less likely to have malaria infection, but the association was not significant in this study population (OR = 0.88, 95% CI 0.26–2.51). Nor was sleeping under a bed net significant association with malaria infection in the owners of bed net population (OR = 0.19, 95% CI 0.00–1.36).

#### Spiritual gathering

Half of the study participants reported attending daily group prayer in a mosque (the daily prayers are held five times a day: before sunrise, noon, afternoon, sunset, and night) 53% (138/261). The data collection time included the holy month of Ramadan, when prayer at a mosque while attending ‘Tarawih’ after dusk and around midnight, may also occur in addition to the normal five times a day prayer. This was measured as “Ramadan which is a nighttime spiritual gathering” and reported by 66% (173/262) of participants. Spiritual gathering and nighttime spiritual gathering in holy month of Ramadan was associated with malaria infection in this study, respectively UOR = 1.82 (95% CI 1.11–3.11) and UOR = 2.03 (95% CI 1.12–3.68).

#### Multivariate analyses

The multivariate logistic regression model was fit with malaria infection as the outcome variable to adjust for potential confounding. Known and measured confounders incorporated were sex, age, socio–economic status, and use of bed net. All variables’ unadjusted and adjusted odds ratios are shown in (Table 2). All variables were included in the final model. Participants who were diagnosed with malaria infection were more likely to have travelled in the last 30 days. Adjusted Odds Ratio AOR: 11.5 (95% CI 4.43–29.9, P-value 0.001). Participants who were involved in Ramadan nighttime spiritual gatherings were more likely to be diagnosed with malaria AOR:3.86 (95% CI 1.10–8.42, P-value 0.009). Men were more likely to have malaria before and after adjustment AOR:4.57 (95% CI 1.43–14.7, P-value 0.01). Among agricultural activities, animal husbandry was significantly associated with malaria AOR = 2.52 (1.09–5.82, P-value 0.031), but farming was not significant after adjusting to sex, age, socio–economic status, travel, participation in spiritual gatherings, and use of bed net.

**Table 2:** Factors associated with malaria diagnosis among persons in Baish District, August 2017-January 2019

Characteristics	UAOR	*P-*value	AO	*P-*value
	(95% CI)		(95% CI)	
Travel	12.4	0. 0001	11.5	0. 0001
	(5.16–29.9)		(4.43—29.9)	
Age	0.99	0.761	0.90	0.818
	(0.98–1.02)		(0.38–2.12)	
Sex				
Female	–	–	–	–
Male	2.93	0. 0001	4.57	0.011
	(1.66–5.18)		(1.43–14.7)	
Work in farm	2.11	0.022	0.89	0.797
	(1.11–3.99)		(0.36–2.17)	
Animal husbandry	2.87	0.001	2.52	0.031
	(1.51–5.49)		(1.09–5.82)	
Daily prayer	1.82	0.029	0.28	0.058
	(1.11–3.11)		(0.07–1.04)	
Ramadan prayer	2.03	0.020	3.04	0.032
	(1.12–3.68)		(1.10–8.42)	
Slept under Bed-net the night prior survey	0.19	0.117	0.22	0.160
	(0.02–1.51)		(0.02–1.83)	

Significant differences between case and controls at the 5% level

*UAOR* unadjusted odds ratio, *UOR* adjusted odds ratio

## Discussion

Results from the southern border of Saudi Arabia case–control study revealed that males involved in animal husbandry and travelling outside their home village within Jazan agriculture areas were at higher risk of malaria infection. They also presented malaria clinical symptoms to health facilities in the region. These findings are considered significant, since common human movement patterns within small geographical areas can lead to a higher risk of malaria infection, which can be a barrier to successfully eliminating malaria and further exacerbate the problem in areas where malaria elimination programmes already struggle with cross–border malaria [55–59]. In low–transmission settings, there is an apparent variation in the intensity of malaria transmission among villages due to agroecosystems, vector populations, and interaction with various livelihood activities and social behaviors [60]. While imported malaria is often cited as a significant obstacle in successfully eliminating malaria from the southern region, overnight travel to neighbouring communities and agricultural areas have enabled increased malaria risk in this study similar to other study findings [61–63]. Furthermore, it was reported in a previous study that 22% of the reported malaria cases did not indicate any travel history. There are more than 60 active transmission foci within Jazan. More than two-thirds of the reported malaria cases had a travel history that can be traced to documented active transmission foci in the last 5 years. There is evidence that the risk of acquiring malaria in Jazan and Aseer region are still high.

Increased malaria among men [64–66] and among individuals reporting recent travels [67–70] has been displayed in border areas, among agricultural workers, and in low endemic areas with human movement in elimination settings [13, 15]. The association of malaria risk and travel comes with a caveat. The increased odds of being male travellers and malaria infection might be explained by gender-related behaviour as well as other influences such as environmental, socio-demographic, and behavioural determinants of malaria infection [71]. For example, in Jazan, only males are involved in farming, which prolongs their contact with malaria vectors. More frequent short-term movement into areas of active transmission located in the agricultural areas during risk periods makes males more prone to mosquito bites. Other behaviours such as inconsistent usage of preventive measures like bed nets facilitate malaria infection. The use of LLNs showed protection to people living in malaria endemic areas in several other studies [72]. All participants in the current study did not use LLNs or any form of bed net during their frequent travels. In addition, attendance at spiritual practicing venues (mosques), especially at night, expand their exposure to mosquito bites. Mosques in the region usually have traditional building structure with open space and incomplete roofing. Another important factor was travelling, which is common in this area for trading in local markets, schools, visiting families and friends [73]. In this investigation, travelling was mainly related to work in agriculture and visiting families and friends in farm areas. However, many participants preferred not to share the reason for their travel or their destination. This has been observed in another study in the southern region [73] and is often cited by malaria elimination programme workers.

Also, investigations revealed that animal husbandries were associated with malaria infections is in line with the other studies indicating that agricultural livelihood activities are associated with an increased risk of malaria transmission [74]. Keeping livestock in households may escalate the risk of malaria by attracting more vectors to nearby households, providing additional blood sources to expand the vector population, and creating favorable larval habitat through puddles, hoof prints, and watering sites [50]. This has also been perceived in previous other studies, where communities kept their animals inside their lodgings or slept outside in proximity to the livestock [75, 76]. Yet, proximity to animals has also often been protective by offering alternative blood meal hosts was observed in some settings [77]. Thus, one of the greatest challenges for malaria elimination are vectors that have a dual feeding tendency preference and can feed frequently enough on humans to maintain residual transmission, but also often enough feed on animals to evade mass population suppression with insecticide measures like IRS and LLINs.

Participating in nighttime spiritual gatherings has shown to be associated with the risk of malaria infection. Regular community gatherings at outdoor night–time venues play a role in malaria transmission [7, 10]. This might be related to many contributing factors, such as mosque architecture being predominantly open instead of closed and people gathering outside in the mosque grounds rather than inside a sealed building. Also, the main vector, *An. arabiensis* is a mosquito more prone to feed outdoors and earlier in the evening (the same time when the prayers take place) and is exophilic (outdoor resting) are not subjected to IRS decreasing its effect and impact [78]. It has been documented that malaria transmission can persist in the context of high levels of ITN or IRS coverage and is known as residual transmission [10]. Mosques also serve as community centres and sanctuaries in complex settings with mobile tribal populations, long–term and short-term migrants, and undocumented workers. The mosques usually serve the whole population and are places where people feel safe, so they could be a good venue for providing malaria prevention education or other services.

In this study, the use of ITNs and bed net ownership did not show any statistically significant protective measures against malaria infection. This contradicts the findings from large–scale studies that showed insecticide-treated nets (ITN) have been reported to be a highly effective intervention for malaria control globally [50, 79], which could be explained by the very low numbers of LLINs ownership in the study population. There is a strong need to increase access, distribution of LLINS and health messages to fill the gap in preventions [80, 81]. Taking into consideration the lifestyle and needs of high-risk groups. For example, long-lasting insecticidal hammocks were found to be protective to vectors bite outside the house [82]. Malaria elimination programmes should consider the different types of wall material when using IRS because wall material might influence the efficacy of IRS [83]. In Jazan, there are a wide variety of housing structures. For example, there are traditional houses made of wattle and daub with thatched roofs, and there are houses made of cement blocks and houses made of mud and stone structures [84]. Also, to ensure the end-users accept and adequately use the intervention protocols in sacred spaces spraying the wall with IRS is not recommended [85, 86]. Some promising results indicate that insecticide paints can be effective [87].

## Limitations

Several limitations were noted in this study design and implementation. First, the study is a small case–control in a confined geographical area with exceptional context that provides limited external validity. Second, though the study focused on subjects that have reported to health facilities and followed them through active and proactive case detection, subjects that do not report a home or work address (highly mobile migrant commonly work in the area) and an individual who lives in border village are inaccessible may not have been captured in this study. Third, recall bias cannot be ruled out in reporting travel history and bed net use. However, the travel history was restricted to 30 days, and the bed net use was limited to the night before the survey to minimize the recall bias. Fourth, due to the small sample size in the analysis, no distinction was made between areas of different transmission intensity. However, such a bias might be toward the null hypothesis, and the study showed a strong association between travel and malaria infection. Fifth, the use of RDTs might have led to misclassification bias, but per malaria elimination programme protocol negative all negative RDTs will still have a thin blood slide and all positive cases had a second microscopy reading to be evaluated. Also, all study participants were tested using two different kinds of RDTs. In addition, due to the very low transmission coupled with the ongoing unrest in the area, recruitment took a long time; the study continued over several transmission seasons missing temporal risk and was confounded by climatic factors. Finally, the small sample size limited the study’s statistical power, leading to a wide confidence interval. The study did not reach the estimated sample size due to many logistical reasons, limiting statistical power.

## Conclusion

Being male, one’s travel history and employment in animal husbandry are risk factors for malaria infection in low transmission settings in agricultural border areas. The findings suggest that males who frequently travelled overnight may have contributed to sustained local transmission and need to be addressed by intervention activities that can benefit them as traditional control measures have limited efficacy. This finding specifically might help the national malaria elimination programme increase intervention coverage amongst high–risk groups in active transmission areas to help in shortening the tale of malaria elimination.

## Data Availability

All the datasets are available on reasonable request to the corresponding author.
